# Supplementary material for: A structural equation model predicts chronic wound healing time using patient characteristics and wound microbiome composition
Source: Wound Repair Regen. 2025 Feb 17;33(1):e70004. doi: 10.1111/wrr.70004 (PMC11831583; doi:10.1111/wrr.70004)
Supplement: Supplementary file 1 — Data S1 [file WRR-33-0-s001.docx]

**SUPPLEMENTARY TABLES**

Supplementary Table 1: Patient demographics and wound characteristics by wound type. Cell values are either means with parenthetical standard deviations, or n with parenthetical percent of total n. The correlation or cooccurrence with the microbiome latent variable is shown depending on the defined relationship in the structural equation model. Values under Slough, Exudate, Percent Granulation, and Edema are the transformed scores according to the TIME-H comorbidity score defined in the methods section.

|  | Decubitus Ulcer,  N = 42 | Diabetic Foot Ulcer,  N = 105 | Atypical Wound,  N = 385 | Venous Leg Ulcer,  N = 33 | Correlation with Shorter Healing Time Microbiome Variable |
| --- | --- | --- | --- | --- | --- |
| Duration (Days) | 293 (297) | 464 (568) | 399 (535) | 271 (330) | -0.6343 |
| Volume |  |  |  |  | -0.1723 |
| Length (cm) | 0.85 (1.82) | 0.48 (0.80) | 0.56 (1.12) | 0.75 (1.52) |  |
| Width (cm) | 0.82 (1.66) | 0.41 (0.53) | 0.54 (1.12) | 0.74 (1.44) |  |
| Depth (cm) | 0.33 (0.72) | 0.19 (0.14) | 0.22 (0.30) | 0.22 (0.29) |  |
| Slough |  |  |  |  | -0.4931 |
| 0 | 26 (62%) | 78 (74%) | 286 (74%) | 23 (70%) |  |
| 1 | 12 (29%) | 26 (25%) | 87 (23%) | 10 (30%) |  |
| 2 | 4 (9.5%) | 1 (1.0%) | 12 (3.1%) | 0 (0%) |  |
| Exudate |  |  |  |  | -0.3334 |
| 0 | 17 (40%) | 58 (55%) | 199 (52%) | 20 (61%) |  |
| 1 | 16 (38%) | 31 (30%) | 134 (35%) | 7 (21%) |  |
| 2 | 9 (21%) | 16 (15%) | 52 (14%) | 6 (18%) |  |
| Percent Granulation |  |  |  |  | 0.0934 |
| 0 | 24 (57%) | 70 (67%) | 242 (63%) | 29 (88%) |  |
| 1 | 1 (2.4%) | 0 (0%) | 6 (1.6%) | 0 (0%) |  |
| 2 | 17 (40%) | 35 (33%) | 137 (36%) | 4 (12%) |  |
| Edema |  |  |  |  | -0.3659 |
| 0 | 24 (57%) | 78 (74%) | 240 (62%) | 22 (67%) |  |
| 1 | 12 (29%) | 25 (24%) | 125 (32%) | 11 (33%) |  |
| 2 | 6 (14%) | 2 (1.9%) | 20 (5.2%) | 0 (0%) |  |

Supplementary Table 2: The table shows the number of wounds each of the species were found in. All wounds counted in the table were not polymicrobial. The table has also been facetted by the wound type.

| **Species** | **Overall,**  **N = 68** | **Decubitus Ulcer,**  **N = 3** | **Diabetic Foot Ulcer,**  **N = 11** | **Atypical Wound,**  **N = 49** | **Venous Leg Ulcer,**  **N = 5** |
| --- | --- | --- | --- | --- | --- |
| *Burkholderia cepacia* | 1 (1.5%) | 0 (0%) | 0 (0%) | 0 (0%) | 1 (20%) |
| *Corynebacterium jeikeium* | 1 (1.5%) | 0 (0%) | 1 (9.1%) | 0 (0%) | 0 (0%) |
| *Corynebacterium striatum* | 5 (7.4%) | 0 (0%) | 1 (9.1%) | 4 (8.2%) | 0 (0%) |
| *Enterococcus faecalis* | 1 (1.5%) | 0 (0%) | 0 (0%) | 1 (2.0%) | 0 (0%) |
| *Escherichia coli* | 1 (1.5%) | 0 (0%) | 0 (0%) | 1 (2.0%) | 0 (0%) |
| *Klebsiella aerogenes* | 1 (1.5%) | 0 (0%) | 0 (0%) | 1 (2.0%) | 0 (0%) |
| *Marinobacter hydrocarbonoclasticus* | 1 (1.5%) | 0 (0%) | 1 (9.1%) | 0 (0%) | 0 (0%) |
| *Pseudomonas aeruginosa* | 8 (12%) | 0 (0%) | 1 (9.1%) | 6 (12%) | 1 (20%) |
| *Staphylococcus aureus* | 32 (47%) | 3 (100%) | 7 (64%) | 19 (39%) | 3 (60%) |
| *Staphylococcus epidermidis* | 8 (12%) | 0 (0%) | 0 (0%) | 8 (16%) | 0 (0%) |
| *Staphylococcus lugdunensis* | 2 (2.9%) | 0 (0%) | 0 (0%) | 2 (4.1%) | 0 (0%) |
| *Staphylococcus pseudintermedius* | 1 (1.5%) | 0 (0%) | 0 (0%) | 1 (2.0%) | 0 (0%) |
| *Streptococcus agalactiae* | 4 (5.9%) | 0 (0%) | 0 (0%) | 4 (8.2%) | 0 (0%) |
| *Streptococcus anginosus* | 1 (1.5%) | 0 (0%) | 0 (0%) | 1 (2.0%) | 0 (0%) |
| *Streptococcus dysgalactiae* | 1 (1.5%) | 0 (0%) | 0 (0%) | 1 (2.0%) | 0 (0%) |

Supplementary Table 3: List of the 66 parceled species incidence per wound type. Additionally, the species correlation with wound healing time and the respective parcel each species was included in is shown. The species pearson correlation with healing time was calculated during parceling procedure defined in the methods.

| **Species** | **Overall, N = 565** | **Decubitus Ulcer,**  **N = 42** | **Diabetic Foot Ulcer,**  **N = 105** | **Atypical Wound, N = 385** | **Venous Leg Ulcer,**  **N = 33** | **Healing Time Pearson Correlation** | **Parcel** |
| --- | --- | --- | --- | --- | --- | --- | --- |
| *Achromobacter xylosoxidans* | 3 (0.5%) | 0 (0%) | 1 (1.0%) | 2 (0.5%) | 0 (0%) | -0.0396 | p2 |
| *Acinetobacter lwoffii* | 4 (0.7%) | 2 (4.8%) | 0 (0%) | 2 (0.5%) | 0 (0%) | -0.0358 | p1 |
| *Acinetobacter radioresistens* | 4 (0.7%) | 0 (0%) | 0 (0%) | 3 (0.8%) | 1 (3.0%) | -0.0406 | p1 |
| *Actinobaculum massiliense* | 5 (0.9%) | 2 (4.8%) | 2 (1.9%) | 1 (0.3%) | 0 (0%) | -0.0234 | p2 |
| *Actinomyces neuii* | 14 (2.5%) | 1 (2.4%) | 5 (4.8%) | 7 (1.8%) | 1 (3.0%) | -0.0404 | p0 |
| *Actinotignum schaalii* | 3 (0.5%) | 0 (0%) | 1 (1.0%) | 2 (0.5%) | 0 (0%) | -0.0272 | p0 |
| *Alcaligenes faecalis* | 6 (1.1%) | 1 (2.4%) | 2 (1.9%) | 3 (0.8%) | 0 (0%) | -0.0271 | p2 |
| *Anaerococcus hydrogenalis* | 30 (5.3%) | 2 (4.8%) | 8 (7.6%) | 17 (4.4%) | 3 (9.1%) | -0.0634 | p0 |
| *Anaerococcus lactolyticus* | 38 (6.7%) | 7 (17%) | 7 (6.7%) | 23 (6.0%) | 1 (3.0%) | -0.0580 | p1 |
| *Anaerococcus octavius* | 10 (1.8%) | 0 (0%) | 0 (0%) | 10 (2.6%) | 0 (0%) | -0.0464 | p0 |
| *Anaerococcus prevotii* | 26 (4.6%) | 1 (2.4%) | 4 (3.8%) | 18 (4.7%) | 3 (9.1%) | -0.0541 | p1 |
| *Bacteroides dorei* | 2 (0.4%) | 0 (0%) | 0 (0%) | 2 (0.5%) | 0 (0%) | -0.0253 | p2 |
| *Bacteroides fragilis* | 13 (2.3%) | 1 (2.4%) | 0 (0%) | 12 (3.1%) | 0 (0%) | -0.0594 | p0 |
| *Bacteroides ovatus* | 2 (0.4%) | 1 (2.4%) | 0 (0%) | 1 (0.3%) | 0 (0%) | -0.0247 | p0 |
| *Bacteroides pyogenes* | 4 (0.7%) | 0 (0%) | 0 (0%) | 4 (1.0%) | 0 (0%) | -0.0205 | p2 |
| *Brevibacterium luteolum* | 3 (0.5%) | 0 (0%) | 0 (0%) | 3 (0.8%) | 0 (0%) | -0.0372 | p1 |
| *Brevundimonas nasdae* | 2 (0.4%) | 0 (0%) | 0 (0%) | 2 (0.5%) | 0 (0%) | -0.0259 | p1 |
| *Campylobacter ureolyticus* | 12 (2.1%) | 2 (4.8%) | 3 (2.9%) | 7 (1.8%) | 0 (0%) | -0.0470 | p2 |
| *Clostridium bolteae* | 2 (0.4%) | 1 (2.4%) | 0 (0%) | 1 (0.3%) | 0 (0%) | -0.0278 | p1 |
| *Corynebacterium afermentans* | 4 (0.7%) | 1 (2.4%) | 0 (0%) | 3 (0.8%) | 0 (0%) | -0.0276 | p1 |
| *Corynebacterium amycolatum* | 12 (2.1%) | 3 (7.1%) | 2 (1.9%) | 7 (1.8%) | 0 (0%) | -0.0288 | p0 |
| *Corynebacterium auriscanis* | 3 (0.5%) | 0 (0%) | 1 (1.0%) | 2 (0.5%) | 0 (0%) | -0.0398 | p0 |
| *Corynebacterium confusum* | 2 (0.4%) | 0 (0%) | 0 (0%) | 2 (0.5%) | 0 (0%) | -0.0239 | p2 |
| *Corynebacterium fournierii* | 2 (0.4%) | 0 (0%) | 0 (0%) | 2 (0.5%) | 0 (0%) | -0.0214 | p2 |
| *Corynebacterium pseudodiphtheriticum* | 4 (0.7%) | 1 (2.4%) | 1 (1.0%) | 2 (0.5%) | 0 (0%) | -0.0488 | p0 |
| *Corynebacterium striatum* | 60 (11%) | 1 (2.4%) | 15 (14%) | 38 (9.9%) | 6 (18%) | -0.0323 | p0 |
| *Corynebacterium tuscaniense* | 2 (0.4%) | 0 (0%) | 0 (0%) | 2 (0.5%) | 0 (0%) | -0.0288 | p2 |
| *Cutibacterium acnes* | 12 (2.1%) | 1 (2.4%) | 0 (0%) | 10 (2.6%) | 1 (3.0%) | -0.0554 | p0 |
| *Dialister micraerophilus* | 6 (1.1%) | 0 (0%) | 0 (0%) | 6 (1.6%) | 0 (0%) | -0.0334 | p0 |
| *Dialister pneumosintes* | 5 (0.9%) | 0 (0%) | 1 (1.0%) | 4 (1.0%) | 0 (0%) | -0.0512 | p0 |
| *Escherichia coli* | 60 (11%) | 6 (14%) | 7 (6.7%) | 43 (11%) | 4 (12%) | -0.0308 | p1 |
| *Faecalibacterium prausnitzii* | 2 (0.4%) | 0 (0%) | 1 (1.0%) | 1 (0.3%) | 0 (0%) | -0.0228 | p2 |
| *Fusobacterium canifelinum* | 5 (0.9%) | 0 (0%) | 3 (2.9%) | 2 (0.5%) | 0 (0%) | -0.0445 | p2 |
| *Fusobacterium nucleatum* | 26 (4.6%) | 1 (2.4%) | 7 (6.7%) | 18 (4.7%) | 0 (0%) | -0.0230 | p1 |
| *Fusobacterium periodonticum* | 6 (1.1%) | 0 (0%) | 2 (1.9%) | 4 (1.0%) | 0 (0%) | -0.0203 | p1 |
| *Gemella morbillorum* | 7 (1.2%) | 0 (0%) | 4 (3.8%) | 3 (0.8%) | 0 (0%) | -0.0303 | p1 |
| *Klebsiella aerogenes* | 2 (0.4%) | 0 (0%) | 0 (0%) | 2 (0.5%) | 0 (0%) | -0.0314 | p1 |
| *Klebsiella oxytoca* | 6 (1.1%) | 0 (0%) | 0 (0%) | 6 (1.6%) | 0 (0%) | -0.0298 | p0 |
| *Klebsiella pneumoniae* | 10 (1.8%) | 1 (2.4%) | 1 (1.0%) | 8 (2.1%) | 0 (0%) | -0.0310 | p2 |
| *Morganella morganii* | 15 (2.7%) | 3 (7.1%) | 3 (2.9%) | 8 (2.1%) | 1 (3.0%) | -0.0394 | p1 |
| *Pasteurella dagmatis* | 2 (0.4%) | 0 (0%) | 0 (0%) | 2 (0.5%) | 0 (0%) | -0.0283 | p0 |
| *Pasteurella multocida* | 6 (1.1%) | 1 (2.4%) | 1 (1.0%) | 4 (1.0%) | 0 (0%) | -0.0453 | p2 |
| *Peptoniphilus harei* | 35 (6.2%) | 5 (12%) | 6 (5.7%) | 23 (6.0%) | 1 (3.0%) | -0.0447 | p2 |
| *Peptostreptococcus anaerobius* | 16 (2.8%) | 1 (2.4%) | 2 (1.9%) | 11 (2.9%) | 2 (6.1%) | -0.0376 | p0 |
| *Porphyromonas bennonis* | 39 (6.9%) | 4 (9.5%) | 7 (6.7%) | 27 (7.0%) | 1 (3.0%) | -0.0329 | p2 |
| *Porphyromonas cangingivalis* | 4 (0.7%) | 0 (0%) | 1 (1.0%) | 3 (0.8%) | 0 (0%) | -0.0399 | p2 |
| *Porphyromonas levii* | 18 (3.2%) | 3 (7.1%) | 4 (3.8%) | 11 (2.9%) | 0 (0%) | -0.0445 | p1 |
| *Porphyromonas somerae* | 24 (4.2%) | 3 (7.1%) | 1 (1.0%) | 19 (4.9%) | 1 (3.0%) | -0.0513 | p1 |
| *Prevotella bergensis* | 9 (1.6%) | 2 (4.8%) | 0 (0%) | 7 (1.8%) | 0 (0%) | -0.0497 | p2 |
| *Prevotella corporis* | 6 (1.1%) | 2 (4.8%) | 1 (1.0%) | 3 (0.8%) | 0 (0%) | -0.0213 | p0 |
| *Prevotella timonensis* | 27 (4.8%) | 5 (12%) | 2 (1.9%) | 20 (5.2%) | 0 (0%) | -0.0205 | p1 |
| *Proteus mirabilis* | 28 (5.0%) | 2 (4.8%) | 5 (4.8%) | 19 (4.9%) | 2 (6.1%) | -0.1191 | p2 |
| *Proteus vulgaris* | 2 (0.4%) | 0 (0%) | 0 (0%) | 1 (0.3%) | 1 (3.0%) | -0.0238 | p2 |
| *Staphylococcus arlettae* | 2 (0.4%) | 0 (0%) | 0 (0%) | 2 (0.5%) | 0 (0%) | -0.0244 | p2 |
| *Staphylococcus aureus* | 195 (35%) | 14 (33%) | 35 (33%) | 133 (35%) | 13 (39%) | -0.1057 | p1 |
| *Staphylococcus capitis* | 16 (2.8%) | 1 (2.4%) | 3 (2.9%) | 11 (2.9%) | 1 (3.0%) | -0.0473 | p0 |
| *Staphylococcus caprae* | 3 (0.5%) | 0 (0%) | 2 (1.9%) | 1 (0.3%) | 0 (0%) | -0.0208 | p0 |
| *Staphylococcus cohnii* | 9 (1.6%) | 1 (2.4%) | 3 (2.9%) | 5 (1.3%) | 0 (0%) | -0.0468 | p1 |
| *Staphylococcus pseudintermedius* | 4 (0.7%) | 0 (0%) | 2 (1.9%) | 2 (0.5%) | 0 (0%) | -0.0424 | p0 |
| *Stenotrophomonas maltophilia* | 9 (1.6%) | 0 (0%) | 2 (1.9%) | 6 (1.6%) | 1 (3.0%) | -0.0365 | p1 |
| *Streptococcus dysgalactiae* | 18 (3.2%) | 1 (2.4%) | 2 (1.9%) | 14 (3.6%) | 1 (3.0%) | -0.0396 | p1 |
| *Streptococcus intermedius* | 6 (1.1%) | 1 (2.4%) | 0 (0%) | 5 (1.3%) | 0 (0%) | -0.0569 | p2 |
| *Streptococcus mitis* | 17 (3.0%) | 2 (4.8%) | 4 (3.8%) | 11 (2.9%) | 0 (0%) | -0.0394 | p2 |
| *Streptococcus oralis* | 6 (1.1%) | 0 (0%) | 0 (0%) | 5 (1.3%) | 1 (3.0%) | -0.0418 | p0 |
| *Streptococcus pyogenes* | 4 (0.7%) | 0 (0%) | 3 (2.9%) | 1 (0.3%) | 0 (0%) | -0.0214 | p1 |
| *Terrahaemophilus aromaticivorans* | 5 (0.9%) | 0 (0%) | 1 (1.0%) | 4 (1.0%) | 0 (0%) | -0.0343 | p0 |
